# Glucagon-like peptide-1 receptor agonist use and clinical outcomes after posterior cervical spinal fusion for degenerative pathologies: A large cohort retrospective analysis

**DOI:** 10.1016/j.xnsj.2026.100873

**Published:** 2026-03-01

**Authors:** Christian Rajkovic, Ankita Jain, Mahnoor Shafi, Oserekpamen Omobhude, Tej Azad, Austin Carpenter, Timothy F. Witham, Merritt D. Kinon, John V. Wainwright

**Affiliations:** a School of Medicine, New York Medical College, Valhalla, NY, United States; b Department of Neurosurgery, Westchester Medical Center, 100 Woods Road, 4th Floor, Valhalla, NY 10595, United States; c Department of Neurosurgery, Johns Hopkins Medicine, Baltimore, MD, United States

**Keywords:** Spine surgery, Cervical spinal fusion, Pseudoarthrosis, Glucagon-like peptide-1, Semaglutide, Liraglutide

## Abstract

**Background:**

Glucagon-like peptide-1 receptor agonists (GLP-1RAs) have emerged as potentially impactful agents in improving spinal fusion outcomes. While several recent studies have investigated the effect of these agents on lumbar spinal procedures, the current evidence of the impact of these agents on cervical spinal fusion is limited. This study aims to investigate the impact of GLP-1RA use on perioperative clinical outcomes of posterior cervical spinal fusion.

**Methods:**

This analysis queried the TriNetX Research Network to identify patients receiving posterior cervical spinal fusion for degenerative pathology. Patients on GLP-1RAs for at least 12 weeks prior to the surgery were compared to patients who were never on GLP-1RAs. Propensity score matching (1:1) was performed for preoperative clinical characteristics and prior spine procedures. Investigated outcomes included postoperative pseudoarthrosis, readmission, reoperation, cervicalgia, radiculopathy, myelopathy, mortality, cerebrospinal fluid leak, infection, and implant failure at 1-year follow-up.

**Results:**

A total of 737 patients with preoperative GLP-1RA use and 18,882 patients with no GLP-1RA use were identified. Prior to propensity score matching, patients on GLP-1RAs had a significantly higher risk of postoperative radiculopathy (p<.001), infection (p=.013), and readmission (p<.001), with a significantly reduced risk of pseudoarthrosis (p=.002). After propensity score matching, the risk of radiculopathy remained significantly elevated in the GLP-1RA cohort (hazard ratio: 1.367, 95% CI: 1.110–1.684, p=.003). The incidence of pseudoarthrosis remained significantly reduced in the GLP-1RA cohort (2.7% vs. 5.2%, p=.019), while a reduced risk of pseudoarthrosis trended toward significance (hazard ratio: 0.586, 95% CI: 0.336–1.022, p=.057).

**Conclusions:**

Preoperative GLP-1RA use was associated with increased postoperative risk of radiculopathy and decreased rates of pseudoarthrosis. The findings from this investigation may help optimize medical and surgical decision-making for posterior cervical spinal fusion patients on GLP-1 agonists.

## Background

Despite advances in surgical techniques and perioperative care, optimizing outcomes and reducing complications in cervical spinal fusion remain significant challenges in an increasingly aging and medically complex patient population [[1], [2], [3]]. Posterior cervical fusion, specifically, is associated with an increased overall complication rate in comparison to anterior cervical discectomy and fusion, particularly in patients with severe preoperative comorbidities [4]. Glucagon-like peptide-1 receptor agonists (GLP-1RAs), primarily used for glycemic control in type 2 diabetes mellitus and weight management, have garnered attention for their prospective impact on spinal surgery outcomes. Therefore, in the context of cervical spinal fusion, where comorbidities like diabetes and obesity are associated with degenerative spinal conditions and elevated complication rates, the role of GLP-1RA in perioperative patient management warrants investigation [5,6].

While several retrospective studies have investigated perioperative outcomes associated with GLP-1RA use prior to spinal fusion, the current body of evidence suggesting any benefit or risk to fusion or clinical outcomes is heterogeneous and limited, specifically for posterior cervical fusion [[7], [8], [9]]. Only one study evaluating exclusively posterior cervical fusion and GLP-1RA currently exists in the literature [10], which observed a negative association with successful fusion, contradicting a potential positive association with fusion success that has been observed in several studies investigating lumbar fusion and GLP-1RA [7,11]. Further, many prior studies only evaluate a single GLP-1RA agent, potentially limiting the generalizability of the impact of an increasingly prevalent medication class on spinal fusion [7,8].

Therefore, a gap in knowledge currently exists examining the impact of GLP-1RA use on posterior cervical fusion. This retrospective cohort study aims to explore the impact of GLP-1RA on complications following posterior cervical spinal fusion for degenerative pathologies of the spine, contributing to the growing body of literature on optimizing care in this patient population. Cases will be investigated using the TriNetX database, enabling long-term assessment of patient outcomes in a large sample-size cohort of patients on GLP-1RA with propensity score-matched comorbidity statuses to non-GLP-1RA control cases of posterior cervical fusion.

## Methods

### Study design

This study queried the TriNetX Research Network, a deidentified patient database with more than 107 participating healthcare organizations across 19 countries on July 31, 2025 [12]. Data in the TriNetX Research Network was fully deidentified according to Section §164.514(a) of the Health Insurance Portability and Accountability Act (HIPAA) Privacy Rule, and therefore, internal review board (IRB) oversight for this study was exempted, as per Johns Hopkins IRB #00265490. Patients receiving posterior cervical spinal fusion for degenerative pathology were identified from this database using relevant Current Procedural Terminology (CPT) and International Classification of Disease (ICD) codes. Patients with traumatic or neoplastic indications for posterior cervical fusion were excluded using relevant ICD coding. Two cohorts were then identified from this query: (1) a cohort with GLP-1RA use at least 12 weeks prior to surgery, and (2) a cohort with no GLP-1RA use prior to or following surgery. A 12-week preoperative window of GLP-RA use was chosen based on effective dosing timelines described in currently published randomized control trials of GLP-1RA use [13]. The anatomic therapeutic chemical code A10BJ was used to identify GLP-1RA class drugs, and the following RxNorm concept unique identifiers were used to identify specific GLP-1RA: 1991302 for semaglutide, 60548 for exenatide, 2601723 for tirzepatide, 1551291 for dulaglutide, and 475968 for liraglutide. The index event for this study was labeled the day of the posterior cervical spinal fusion, as defined by simultaneous coding with relevant CPT codes for procedures and ICD codes for diagnosis. A full list of investigated CPT and ICD codes for cervical spine surgery is outlined in Supplementary File 1.

### Baseline characteristics and propensity-based matching

To minimize confounding factors between the GLP-1RA and non-GLP-1RA cohorts, propensity score matching (1:1) with logistic regression was performed using the TriNetX platform for the following demographics: age at index procedure, race, sex, ethnicity, body mass index (BMI), and HbA1c. Age was considered a continuous variable, while race, sex, and ethnicity were considered categorical variables. BMI was stratified as a categorical variable with five categories: 0–18.5, 18.5–4.9, 25–29.9, 30–34.9, and >35 kg/m^2^. HbA1c was stratified as a categorical variable with three categories: <5.7%, 5.7%–6.4%, and >6.4%. The following comorbidities were also included in the propensity score matching as categorical variables: dyslipidemia, hypertension, diabetes mellitus, current tobacco use, overweight and obesity status, osteoporosis with and without pathological fracture, ischemic heart disease, cerebrovascular disease, heart failure, and chronic kidney disease. Full demographics and comorbidities of each cohort are outlined in Table 1.

**Table 1:** Baseline clinical characteristics of patient cohorts before and after propensity score matching.

### Outcomes

All outcomes were investigated with 1 year of follow-up, and primary outcomes included the following postoperative complications, as defined by appropriate ICD coding: pseudoarthrosis (M96.0), readmission (99221, 99222, 99223), and reoperation (22600, 22590, 22595, 22554, 22551, 22552). Occurrences of postoperative pseudoarthrosis were only investigated if they were coded between 6 months postoperatively and our final 1-year follow-up period to ensure valid clinical assessment of fusion based on reasonable timelines published in the literature [14]. Any instances of postoperative cervicalgia (M54.2), radiculopathy (M54.11, M54.12, or M54.13), myelopathy (M47.11, M47.12, or M47.13), mortality (R99), cerebrospinal fluid leak (G97.0 or G97.4), postoperative infection (T81.4), implant failure (T84) and postoperative GLP-1RA use (A10BJ, 1991302, 60548, 2601723, 1551291, 475968) were investigated as secondary outcomes. All postoperative complications were considered only if they occurred following the index cervical procedure.

### Statistical analysis

All statistical analyses were performed using TriNetX’s Advanced Analytics Platform, using R version 3.4.4 (R Foundation for Statistical Computing) and Python version 3.6.5 (Python Software Foundation, Centrum voor Wiskunde en Informatica). Each outcome was analyzed using Cox proportional hazard models to compare GLP-1 and non-GLP-1 cohorts for each investigated outcome. All variables with counts less than 11 in each cohort were defined as ≤10. Differences in outcomes between cohorts were analyzed using Chi-squared testing with a significance threshold set at *α*<0.05. Kaplan–Meier graphs were generated to compare the occurrence of primary investigated outcome events across GLP-1RA and non-GLP-1RA cohorts (Figure).

**Figure:**
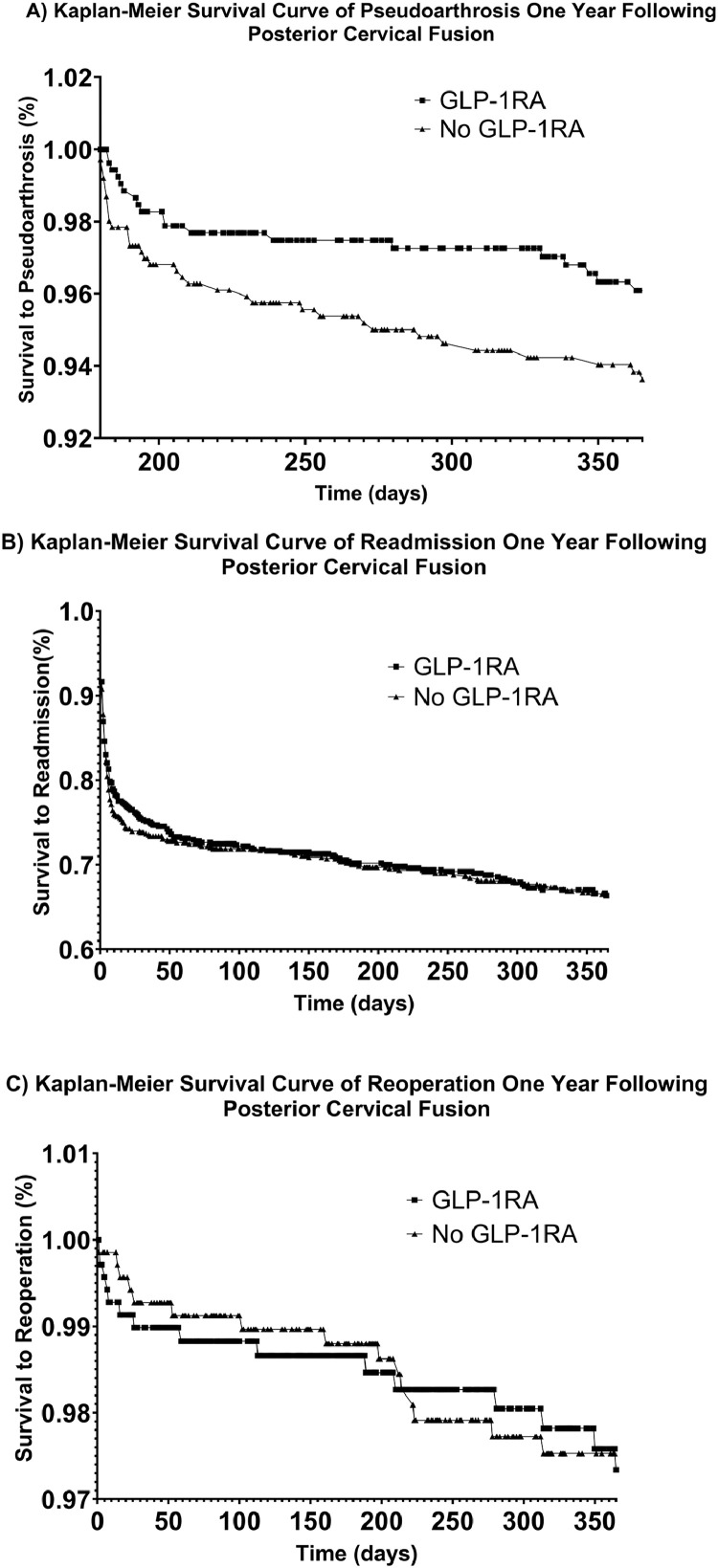
Kaplan–Meier survival curve of postoperative complications for patients receiving cervical spine surgery. The following postoperative complications were investigated as primary outcomes in the GLP-1RA cohort and non-GLP-1RA cohort: (Left) pseudoarthrosis, (Middle) readmission, and (Right) reoperation. Rates of readmission and reoperation were similar over a 1-year follow-up period, while a reduced risk of pseudoarthrosis was trended toward significance (p=.057) in the GLP-1RA cohort compared to the non-GLP-1RA cohort. GLP-1RA, glucagon-like peptide-1 receptor agonist.

## Results

### Baseline characteristics

A total of 19,619 patients who received posterior cervical spinal fusion for degenerative pathology were identified in the TriNetX Research Network. Of this total, 18,882 (96.2%) were recorded to never have had any GLP-1RA use before or after their index procedure, and 737 (3.8%) of these patients were recorded to have used GLP-1RAs at least 12 weeks prior to their index procedure. Baseline clinical characteristics of each cohort are reported in Table 1. Initially, patients in the GLP-1RA cohort were significantly older at index procedure (63.4±9.4 years old vs. 62.3±13.0 years old, p=.018), had significantly more women (54.0% vs. 43.1%, p<.001), and had significantly higher average HbA1c (6.9±1.5 vs. 6.0±1.2, p<.001) and average BMI (33.7±6.6 vs. 29.0±6.4, p<.001). Further, every investigated comorbidity and prior spinal procedure code was observed to have a significantly higher proportion of patients in the GLP-1RA cohort compared to the non-GLP-1RA cohort. Following propensity score matching, patients in the GLP-1RA cohort had a significantly higher proportion of patients with normal BMIs than the non-GLP-1RA cohort (12.8% vs. 9.2%, p=.032); however, the average BMI of each cohort was statistically similar (33.5±6.6 kg/m^2^ vs. 34.0±6.7 kg/m^2^, respectively, p=.211). All remaining investigated preoperative baseline clinical variables and comorbidities were statistically similar.

### Clinical outcomes

The incidences of all investigated outcomes before and after matching are reported in Table 2. Before propensity-score matching, the GLP-1RA cohort had significantly more postoperative occurrences of radiculopathy (p<.001), infection (p=.017), and readmission (p<.001) compared to the non-GLP-1RA cohort and significantly fewer postoperative occurrences of pseudoarthrosis (p<.001) compared to the non-GLP-1RA cohort. After propensity-score matching, the GLP-1RA cohort continued to have significantly lower pseudoarthrosis rates than the non-GLP-1RA cohort (2.7% vs. 5.2%, p=.019), and no significant difference was observed in postoperative readmission (p=.774) or reoperation rate (p=1.000) between the two cohorts. Incidence of postoperative radiculopathy was also observed to be significantly higher in the matched GLP-1RA cohort compared to the matched non-GLP-1RA cohort (28.6% vs. 22.8%, p=.014). Following posterior cervical fusion, 40.4% of patients in the GLP-1RA matched cohort received a subsequent GLP-1RA prescription with an average of 3±3 prescriptions over the 1-year follow-up period.

**Table 2:** Incidences of investigated outcomes in both cohorts before and after propensity score matching.

Results of measures of association analysis and Cox proportional hazard analysis comparing the GLP-1RA cohort to the non-GLP-1RA cohort over a 1-year follow-up period are reported in Table 3 with respective odds ratios and hazard ratios (HRs). Similar to the results of chi-square comparisons of outcomes between each cohort, significantly elevated risks of radiculopathy (p<.001), infection (p=.013), and readmission (p<.001), as well as a reduced risk of pseudarthrosis (p=.002) was associated with preoperative GLP-1RA use before propensity score matching. After propensity-score matching, a reduced risk of pseudoarthrosis, which trended toward significance, was observed with preoperative GLP-1RA use (HR: 0.586, 95% CI: 0.336–1.022, p=.057). Hazard analysis revealed similar risk of both reoperation (HR: 1.073, 95% CI: 0.524–2.196, p=.847) and readmission (HR: 0.989, 95% CI: 0.821–1.191, p=.910) in the GLP-1RA and non-GLP-1RA cohorts. Preoperative GLP-1RA use was also associated with a significantly increased risk of radiculopathy over a 1-year follow-up period compared to no preoperative GLP-1RA use prior to posterior cervical fusion (HR: 1.367, 95% CI: 1.110–1.684, p=.003). Kaplan–Meier survival curves investigating the primary outcomes of pseudoarthrosis, readmission, and reoperation over a 1-year follow-up period are depicted in Figure.

**Table 3:** Calculated odds ratios and hazard ratios describing the impact of glucagon-like peptide-1 receptor agonist use on investigated outcomes following posterior cervical fusion.

## Discussion

This retrospective cohort study utilized the TriNetX Research Network to analyze perioperative complications in patients undergoing posterior cervical fusion for degenerative pathology, comparing those using GLP-1RA for at least 12 weeks preoperatively to a propensity-score-matched cohort that had never received GLP-1RA. Significantly greater rates of postoperative infection and readmission were observed in the GLP-1RA only prior to propensity score matching, suggesting an expected and observed higher comorbidity burden in that cohort. Following propensity-score matching, a significantly lower incidence of pseudoarthrosis was associated with GLP-1RA use, while a significantly higher incidence and risk of radiculopathy was also observed with GLP-1RA use over a 1-year follow-up period.

This study supplements two prior studies investigating cervical spinal fusion and GLP-1RA use. Vatsia et al. [15] investigated rates of pseudoarthrosis and postoperative infection in multilevel anterior cervical discectomy and fusion and multilevel posterior cervical fusion. Similar to this study, they also observed a significantly decreased pseudoarthrosis rate with preoperative GLP-1RA use and no significant difference in postoperative infection. However, this study is limited in the breadth of outcomes described and the exclusion of single-level procedures investigated in this study of posterior cervical fusion. A study by Ng et al. [10] similarly investigated cases of posterior cervical fusion with perioperative semaglutide use from 2010 to 2022 and observed elevated risk of pseudoarthrosis and dysphagia with GLP-1RA use. This study describes investigation of posterior cervical fusion and GLP-1RA in a larger cohort with more current cases and inclusion of more diverse GLP-1RA agents relevant to the rapidly increasing prescription of this medication class.

### Pseudoarthrosis and preoperative GLP-1RAs

The impact of GLP-1RAs on pseudoarthrosis has been investigated in several retrospective studies of spinal fusion procedures, with the vast majority of these studies also observing a decrease in pseudoarthrosis with preoperative GLP-RA use, even while controlling for HbA1c and BMI, as also seen in our study [10,11,[15], [16], [17]]. The reason for this phenomenon is thought to be due to the direct positive influence of GLP-1RA on bone remodeling and vascular endothelial function at the fusion site [7]. In vitro studies have observed the presence of GLP-1 receptors on osteoblastic precursor cells and the prolonged survival of these cells with treatment using GLP-1RA [18]. Further, the control of microvascular disease with these agents may improve microvascular infiltration of formed fusion masses and, therefore, improve osteogenesis of the fusion mass [19]. Adequate nutrition of newly formed bone with neovascularization is critical for continuous bone formation and eventual successful arthrodesis [20].

However, contrary evidence to the benefit of GLP-1RAs on successful spinal fusion also exists in the literature. A study of semaglutide use prior to posterior cervical fusion cases in the PearlDiver Mariner database from 2010 to 2022 has observed a five times increased risk of pseudoarthrosis with semaglutide use over a 2-year follow-up period, postulating a potential loss of bone density with increased bone resorption in the context of weight loss in these patients [10]. The effect size of the reduced incidence of pseudoarthrosis observed in the GLP-1RA cohort of this study was particularly pronounced to the contrary conclusion, with patients who received GLP-1RA preoperative approximately half as likely to develop postoperative pseudoarthrosis compared to the matched non-GLP-1RA cohort. Therefore, the inclusion of all GLP-1RAs in this more recent retrospective cohort warrants consideration as a significant supplement to the literature investigating spinal arthrodesis rates with GLP-1RAs.

### GLP-1RA effect on radiculopathy

While several studies, including this one, have observed a significant benefit to arthrodesis rates with preoperative GLP-1RAs, suggesting superior clinical improvement in patients on GLP-1RAs, the impact of GLP-1RAs on specific clinical outcomes of spinal fusion, particularly cervical spinal fusion, has not been fully elucidated. Presentations of degenerative pathologies of the cervical spine are often varied, depending on the degree of involvement of myelopathic, radicular, and arthritic forms of disease [21]. Prior studies of cervical spinal fusion have shown that cervical spinal fusion has a strong impact in preventing progression or improving severe myelopathic symptoms, with weaker evidence for surgical improvement of pain or radicular symptoms [22,23].

GLP-1 receptors are widely expressed in the central nervous system, including the spinal cord, where their activation modulates neuroinflammation, oxidative stress, and apoptosis [24,25]. Beyond their metabolic effects, GLP-1RAs have been shown to regulate glial function, particularly by attenuating microglial activation and shifting the balance toward an anti-inflammatory phenotype [26]. Systemically, GLP-1RAs promote β-endorphin release and suppress proinflammatory cytokines, such as TNF-α and IL-1β, reducing neuroinflammatory responses that can contribute to postoperative pain and neurologic dysfunction [[26], [27], [28]]. In this study, no difference in postoperative cervicalgia or myelopathy was seen between the GLP-1RA and non-GLP-1RA cohorts, but a significantly elevated risk of postoperative radiculopathy was seen in the GLP-1RA cohort.

Although the systemic health benefits and the direct anti-inflammatory effects of GLP-1RAs have been associated with reduced chronic pain in patients, several examples of worsened pain syndromes with GLP-1RA use exist in the literature. The sympathetic activation associated with GLP-1RA use has also been associated with neural overstimulation and an increased risk of nonarteritic anterior ischemic optic neuropathy [29]. Further, the rapid metabolic effects associated GLP-1RA use have also been implicated in chronic pain and neurologic diseases such as diabetic lumbosacral radiculoplexus neuropathy in patients with rapid HbA1c reduction and common fibular neuropathy in patients with rapid weight loss. Concerning the spine, patients on GLP-1RAs often have reduced lean muscle mass proportional to weight loss and, as a result, may be more likely to have a hampered postoperative recovery and persistent radicular symptoms due to degenerative cervical disease [[30], [31], [32]].

### Limitations

This study has important limitations that ought to be considered. As a retrospective analysis utilizing the TriNetX Research Network, our findings are inherently subject to selection bias and potential inaccuracies in coding. TriNetX utilizes natural language processing algorithms to combine and consolidate relevant reports and assessment to supplement standard ICD-10 and CPT coding to limit errors at the level of data extraction [33]. However, TriNetX is still susceptible to any data input errors due to inherent systematic, clinical, and logistic errors applicable in any electronic health record, and therefore, large database clinical studies. Despite efforts to perform propensity score matching, residual confounding variables may still influence the results, as the dataset does not capture unmeasured clinical, socioeconomic, or patient-reported factors that could impact outcomes.

To minimize the effects of any coding inaccuracies, investigated preoperative clinical characteristics and outcomes were restricted to clinically reasonable timelines of follow-up. Further, the level of granularity in the database considering surgical detail and perioperative medication use, is also limited. While cases were solely restricted to posterior cervical fusion, adjunct biologics and specific bone graft materials could not be investigated and controlled for in each cohort, and thus, represent potential confounders for postoperative pseudoarthrosis. Although previous studies have linked poorer outcomes with a BMI>35 kg/m² and HbA1c>7.0%, we were unable to perform a subgroup analysis for these patients, as they represented a small subset within both cohorts [34,35]. Instead, we adjusted for these patients during propensity score matching to analyze the effect of GLP-1RAs on posterior cervical spinal fusion outcomes independent of patient clinical status.

Finally, the use of electronic health record data limits our ability to account for medication adherence and changes in GLP-1RA use over time postoperatively. However, a period of 12 weeks prior to the index procedure was chosen when filtering for GLP-1RA use to ensure an adequate regimen of GLP-1RAs to influence postoperative outcomes. Further, postoperative GLP-1RA use was also investigated as an outcome to describe adherence to GLP-1RA use after surgery. Despite these limitations, the large-scale nature of this retrospective analysis of more than 20,000 patients provides a robust examination of this clinically important topic.

## Conclusions

This population-based, retrospective cohort study specifically examines the role of GLP-1RA in several postoperative clinical outcomes—including postoperative infection, pseudoarthrosis, cerebrospinal fluid leak, implant failure, mortality, cervicalgia, radiculopathy, myelopathy, reoperation, and readmission—in adult patients undergoing posterior cervical fusion surgeries for degenerative pathologies of the spine. Data analysis demonstrated that patients on GLP-1RAs had a similar safety profile compared to patients not on GLP-1RAs, with a significantly elevated risk of postoperative radiculopathy and a significantly decreased incidence of postoperative pseudoarthrosis. These helpful findings inform spine surgeons on how to optimize medical and surgical decision-making for patients with significant comorbidities on GLP-1RAs undergoing posterior cervical spinal fusion.

## Funding

No funding was used for this study. Dr Timothy Witham owns stock in Augmedics, Inc, is a consultant for Depuy-Synthes Spine, and is on the surgical advisory boards of Augmedics, Inc, Cerapedics, and Kuros Biosciences. Dr Merritt Kinon reports stock and honorarium with Globus Medical, Inc and is a consultant for Sanara MedTech.

## Declarations of competing interests

The remaining authors have no conflicts of interest or sources of funding to disclose.

## Appendix Supplementary materials

Image, application 1
