# Supplementary material for: Glucagon-like peptide-1 receptor agonist use and clinical outcomes after posterior cervical spinal fusion for degenerative pathologies: A large cohort retrospective analysis
Source: N Am Spine Soc J. 2026 Mar 1;26:100873. doi: 10.1016/j.xnsj.2026.100873 (PMC13049663; doi:10.1016/j.xnsj.2026.100873)
Supplement: Supplementary file 1 [file mmc1.docx]

**Supplementary File 1. Coding for Patient Cohort** ICD-10, CPT, and RxCUI codes used to identify patients on GLP-1 agonists who received posterior cervical spinal fusion for degenerative pathology

| **Code** | **Description** |
| --- | --- |
| **Included Spinal Pathologies – ICD-10 Codes** | |
| M50 | Cervical disc disorders |
| M43.3 | Recurrent atlantoaxial dislocation with myelopathy |
| M54.2 | Cervicalgia |
| M43.01 | Spondylolysis, occipito-atlanto-axial region |
| M43.02 | Spondylolysis, cervical region |
| M43.03 | Spondylolysis, cervicothoracic region |
| M43.11 | Spondylolisthesis, occipito-atlanto-axial region |
| M43.12 | Spondylolisthesis, cervical region |
| M43.13 | Spondylolisthesis, cervicothoracic region |
| M47.11 | Other spondylosis with myelopathy, occipito-atlanto-axial region |
| M47.12 | Other spondylosis with myelopathy, cervical region |
| M47.13 | Other spondylosis with myelopathy, cervicothoracic region |
| M47.21 | Other spondylosis with radiculopathy, occipito-atlanto-axial region |
| M47.22 | Other spondylosis with radiculopathy, cervical region |
| M47.23 | Other spondylosis with radiculopathy, cervicothoracic region |
| M48.01 | Spinal stenosis, occipito-atlanto-axial region |
| M48.02 | Spinal stenosis, cervical region |
| M48.03 | Spinal stenosis, cervicothoracic region |
| M54.11 | Radiculopathy, occipito-atlanto-axial region |
| M54.12 | Radiculopathy, cervical region |
| M54.13 | Radiculopathy, cervicothoracic region |
| M47.811 | Spondylosis without myelopathy or radiculopathy, occipito-atlanto-axial region |
| M47.812 | Spondylosis without myelopathy or radiculopathy, cervical region |
| M47.813 | Spondylosis without myelopathy or radiculopathy, cervicothoracic region |
| M47.891 | Other spondylosis, occipito-atlanto-axial region |
| M47.892 | Other spondylosis, cervical region |
| M47.893 | Other spondylosis, cervicothoracic region |
| M53.2X1 | Spinal instabilities, occipito-atlanto-axial region |
| M53.2X2 | Spinal instabilities, cervical region |
| M53.2X3 | Spinal instabilities, cervicothoracic region |
| **Excluded Spinal Pathologies – ICD-10 Codes** | |
| C41.2 | Malignant neoplasm of vertebral column |
| D33.4 | Benign neoplasm of spinal cord |
| C79.51 | Secondary malignant neoplasm of bone |
| S10-S19 | Injuries to the neck |
| S00-S09 | Injuries to the head |
| **Posterior Cervical Spinal Fusion – CPT Codes** | |
| 22590 | Arthrodesis, posterior technique, craniocervical (occiput-C2) |
| 22595 | Arthrodesis, posterior technique, atlas-axis (C1-C2) |
| 22600 | Arthrodesis, posterior or posterolateral technique, single interspace; cervical below C2 segment |
| **GLP-1 Agonists – RxNorm Codes** | |
| ATC:A10BJ | Glucagon-like peptide-1 (GLP-1) analogues |
| 1991302 | Semaglutide |
| 475968 | Liraglutide |
| 1551291 | dulaglutide |
| 60548 | exenatide |
| 2601723 | tirzepatide |
